# Supplementary material for: Causal Relationship between Aging and Anorexia Nervosa: A White-Matter-Microstructure-Mediated Mendelian Randomization Analysis
Source: Biomedicines. 2024 Aug 16;12(8):1874. doi: 10.3390/biomedicines12081874 (PMC11351342; doi:10.3390/biomedicines12081874)
Supplement: Supplementary file 1 [file biomedicines-12-01874-s001.zip › Supplementary Figure/Figure S8/Leaveoneout plot of MR analysis for TL to ubm-b-1661.pdf]

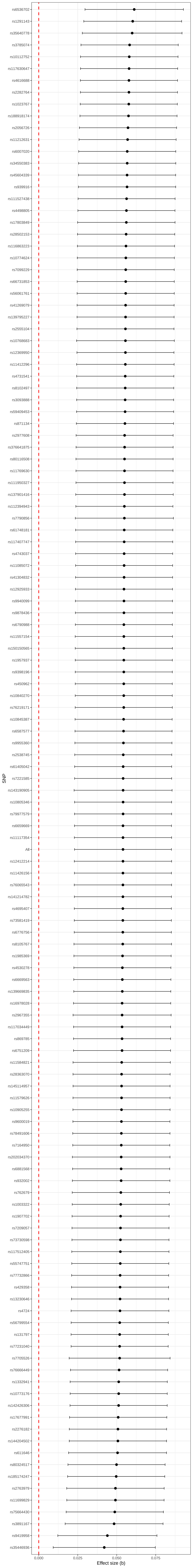
rs6536702
rs1291143
rs35640778
rs3785074
rs10112752
rs117630647
rs4616688
rs2282764
rs1023767
rs188918174
rs2056726
rs11212631
rs6007020
rs34550383
rs45604339
rs939916
rs111527438
rs4498805
rs17803849
rs28502153
rs116863223
rs10774624
rs7099229
rs66731853
rs56061761
rs41269079
rs139795227
rs2555104
rs10768683
rs12369950
rs11412296
rs4731541
rs8102497
rs3093888
rs59409453
rs871134
rs2977608
rs376641875
rs80116508
rs11769630
rs111950327
rs137901416
rs112394943
rs7790856
rs61748181
rs117407747
rs4743037
rs11085072
rs41304832
rs12925933
rs9940099
rs9878436
rs6790988
rs11557154
rs150150565
rs1957937
rs9398196
rs450962
rs10840270
rs76219171
rs10845387
rs6587577
rs9955360
rs2538745
rs61405042
rs7221585
rs143190905
rs10805346
rs79977579
rs6659669
rs11117354
All
rs12412214
rs11426156
rs76065543
rs141214782
rs4695407
rs73581419
rs6776756
rs8105767
rs1985369
rs4530278
rs6669563
rs139669835
rs16978028
rs2967355
rs117034449
rs869785
rs6751209
rs11584821
rs28363070
rs145114957
rs11579626
rs10905255
rs9600019
rs78491606
rs7164950
rs202034370
rs6881568
rs932002
rs762679
rs1003322
rs1907702
rs7209057
rs73730598
rs117512405
rs55747751
rs77732866
rs429358
rs13230646
rs4724
rs56799554
rs131797
rs77231040
rs7705526
rs76666449
rs1332941
rs10773176
rs142426306
rs17677991
rs2276182
rs144204502
rs611646
rs80324517
rs185174247
rs2763979
rs11699829
rs75664430
rs3891167
rs9419958
rs35446936
SNP
0.000
0.025
0.050
0.075
Effect size (b)
